# Academic medical centers as innovation ecosystems to address population –omics challenges in precision medicine

**DOI:** 10.1186/s12967-018-1401-2

**Published:** 2018-02-15

**Authors:** Patrick J. Silva, Valerie M. Schaibley, Kenneth S. Ramos

**Affiliations:** 1 Office of the Senior Vice President Health Sciences, University of Arizona Health Sciences, Drachman Hall, Room B207, 1295 North Martin Avenue, P.O. Box 210202, Tucson, AZ 85721-0202 USA; 2 University of Arizona College of Medicine-Phoenix, 550 E. Van Buren Street, Phoenix, 85004 USA; 3 University of Arizona College of Medicine-Tucson, 1295 North Martin Avenue, Drachman Hall, Room B207, P.O. Box 210202, Tucson, AZ 85721-0202 USA; 4 Center for Applied Genetics and Genomic Medicine, University of Arizona, 1295 North Martin Avenue, Drachman Hall, Room B207, Tucson, AZ 85721-0202 USA

**Keywords:** Precision medicine, Genomics, Biomarker collaboration, Open innovation, Diagnostic technology, Technology transfer, Licensing, Industry alliance, Data ecosystem

## Abstract

While the promise of the Human Genome Project provided significant insights into the structure of the human genome, the complexities of disease at the individual level have made it difficult to utilize –omic information in clinical decision making. Some of the existing constraints have been minimized by technological advancements that have reduced the cost of sequencing to a rate far in excess of Moore’s Law (a halving in cost per unit output every 18 months). The reduction in sequencing costs has made it economically feasible to create large data commons capturing the diversity of disease across populations. Until recently, these data have primarily been consumed in clinical research, but now increasingly being considered in clinical decision- making. Such advances are disrupting common diagnostic business models around which academic medical centers (AMCs) and molecular diagnostic companies have collaborated over the last decade. Proprietary biomarkers and patents on proprietary diagnostic content are no longer driving biomarker collaborations between industry and AMCs. Increasingly the scope of the data commons and biorepositories that AMCs can assemble through a nexus of academic and pharma collaborations is driving a virtuous cycle of precision medicine capabilities that make an AMC relevant and highly competitive. A rebalancing of proprietary strategies and open innovation strategies is warranted to enable institutional precision medicine asset portfolios. The scope of the AMC’s clinical trial and research collaboration portfolios with industry are increasingly dependent on the currency of data, and less on patents. Intrapeneurial support of internal service offerings, clinical trials and clinical laboratory services for example, will be important new points of emphasis at the academic-industry interface. Streamlining these new models of industry collaboration for AMCs are a new area for technology transfer offices to offer partnerships and to add value beyond the traditional intellectual property offering.

## Background

The currency of traditional academic clinical research has been peer-reviewed scholarship. Public investments in science are increasingly requiring researchers to provide other valuable outputs to society as measures of return on investment, including data commons, specimen repositories, patents, and economic impact. Patents are becoming decreasingly important while data and specimen ecosystems are becoming increasingly important. These trends are fundamentally changing the collaboration models that academic medical centers (AMCs) deploy with their clinical affiliates and partners in the biotechnology and biopharma industries. Many institutional policies and administrative processes built for simpler historical collaboration models are proving inadequate to enable large multiparty collaborations that require customized approaches to the management of data, specimens, and patents to balance the unique needs and requirements of public and private interests.

### The Human Genome Project

The Human Genome Project (HGP) represents the largest scale open innovation project in biomedicine to date [1]. The economic impact of the HGP has been difficult to measure in dollars, but the scope of its impact has been profound [2–4]. The economic and intellectual footprint of the HGP has been ubiquitous across science, medicine, and society. A significant expanse of the biomedical research community utilizes sequencing tools, algorithms, and data borne of the HGP. Appropriate or not, genetic sequencing services are being offered direct to consumers for ancestry, wellness, and even medical uses. Genetic testing and biomarkers are guiding the development, regulatory approval, and use of a growing number of new drugs, that were designed themselves based on an understanding of biology enabled by the HGP (Table 1).

**Table 1:** Comprehensive table of FDA approved companion diagnostics and their corresponding drug. Souce: Adapted from FDA.gov on January 6, 2018. http://www.fda.gov/MedicalDevices/ProductsandMedicalProcedures/InVitroDiagnostics/ucm301431.htm

Biomarker (only FDA approved assays are listed)	Tumor type	Therapies guided	NDA	PMA	Analytic method
CD117					
D816V	Aggressive systemic mastocytosis	Imatinib	NDA21335	H140006	PCR
Protein		Imatinib	NDA21335, NDA021588	P040011/S001/S002	IHC
PDGF receptor B					
Rearrangment		Imatinib	NDA21335	H140005	FISH
EGFR receptor					
Exon 19 deletions,T790M	Non-small cell lung	Osimertinib	NDA208065	P120019, S007	PCR
Exon 19 deletions, L858R	Non-small cell lung	Gefitinib, afatinib	NDA206995, NDA201292	P120022/S001	PCR
EGFR receptor protein	Non-small cell lung	Cetuximab, panitumumab	BLA125084, BLA125147	P030044/S001/S002	IHC
Exon 19 deletions, L858R	Non-small cell lung	Erlotinib	NDA021743	P120019/S001/S004	PCR
Exon 19 deletions, L858R	Non-small cell lung	Afatinib	NDA201292	P120022/S001	PCR
Exon 19 deletions, T790 M	Non-small cell lung (circulating tumor)	Erlotinib, osimertinib	NDA208065, NDA021743	P150044, P150047	RTPCR
Exon 19 deletions, L858R	Non-small cell lung	Gefitinib	NDA206955	P170021	NGS
Exon 19 deletions, T790M	Non-small cell lung	Osimertinib	NDA208065	P170021	NGS
Exon 19 deletions, L858R	Non-small cell lung	Erlotinib	NDA021743	P170021	NGS
Exon 19 deletions, L858R	Non-small cell lung	Afatinib	NDA201292	P170021	NGS
Exon 19 deletions, L858R	Non-small cell lung	Gefitinib	206995	P160045	NGS
Her2/Neu					
Gene amplification	Breast cancer	Trastuzumab	BLA103792	P940004	FISH
Gene amplification	Breast cancer	Trastuzumab	BLA103792	P980024/S001/S012	FISH
Gene amplification	Breast cancer	Trastuzumab	BLA103792	P050040/S001/S003	CISH
Gene amplification	Breast cancer, gastric/gastro-esophogeal tumor	Trastuzumab, adotrastuzumab emtansine, pertuzumab	BLA103792, BLA125409	P040005	FISH
Gene amplification: Chrom 17	Breast cancer	Trastuzumab	BLA103792	P100024/S001/S005	CISH
Protein	Breast cancer	Trastuzumab	BLA103792	P090015	IHC
Protein	Breast cancer	Trastuzumab	BLA103792	P990081/S001/S028	IHC
Protein	Breast cancer	Trastuzumab	BLA103792	P040030	IHC
Protein	Breast cancer	Trastuzumab	BLA103792	P980018/S001/S002	IHC
Protein	Breast cancer, gastric/gastroesophogeal Tumor	Trastuzumab, adotrastuzumab-emtansine, pertuzumab	BLA103792, BLA125409	P980018/S001/S002	IHC
Gene amplification	Breast cancer	Trastuzumab	BLA103792	P170021	NGS
Gene amplification	Breast cancer	Pertuzumab	BLA125409	P170021	NGS
Gene amplification	Breast cancer	Adotrastuzumab emtansine	BLA125427	P170021	NGS
KRAS					
Codon 12 and 13 mutations	Colorectal cancer	Cetuximab, panitumumab	BLA125084, BLA125147	P110030, P110027	PCR
	Colorectal cancer	Cetuximab, panitumumab	BLA125084, BLA125147	P140023	PCR
*KRAS* wild-type (absence of mutations in codons 12 and 13)	Colorectal cancer	Cetuximab	BLA125084	P170021	NGS
*RAS* wild-type (absence of mutations in exons 2, 3, and 4) and NRAS wild type (absence of mutations in exons 2, 3, and 4)	Colorectal cancer	Panitumumab	BLA125147	P170021	NGS
56 specific mutations in RAS genes [KRAS (exons 2, 3, and 4) and NRAS (exons 2, 3, and 4)]	Colorectal cancer	Panitumumab	BLA125147	P160038	NGS
BRCA1 and BRCA 2					
Deleterious alterations	Breast cancer	Olaparib	NDA206162	P140020	PCR- > Sanger sequencing
Deleterious alterations	Ovarian cancer	Rucaparib	NDA209115	P160018	NGS
Deleterious alterations	Breast and ovarian cancer	Olaparib	NDA208558	P140020/S012	PCR- > Sanger sequencing
Deleterious alterations	Breast cancer	Rucaparib	NDA209115	P170021	NGS
Deleterious alterations	Breast cancer	Rucaparib	NDA209115	P160018	NGS
Deleterious alterations	Breast cancer	Cobimetinib + vemurafenib	NDA206192	P170021	NGS
PD-1					
Protein	NonSmall Cell Lung	Pembrolizumab	BLA125514/S5	P150013	IHC
Protein	NonSmall cell lung cancer, gastric and gastroesophageal junction adenocarcinoma	Pembrolizumab	sBLA125514/s24	P150013/S006	IHC
Protein	NonSmall Cell Lung	Pembrolizumab	BLA125514 (S008 and S012)	P150013/S001	IHC
ALK					
Protein	NonSmall Cell Lung	Crizotinib	NDA202570	P140025	IHC
Rearrangement	NonSmall Cell Lung	Crizotinib	NDA202570	P110012	FISH
Rearrangement	NonSmall Cell Lung	Crizotinib	NDA202570; NDA 202570/S-021	P170021	NGS
Rearrangement	NonSmall Cell Lung	Alectinib	NDA208434/S-003	P170021	NGS
Rearrangement	NonSmall Cell Lung	Ceritinib	NDA 205755/S-009	P170021	NGS
Protein	NonSmall Cell Lung	Ceritinib	sDNA 205755-09 (NDA supplement)	P140025/S005	IHC
BRAF					
V600E/K	Melanoma	Tramatenib, dabrafenib	NDA204114, NDA202806	P120014	PCR
V600E	Melanoma	Vemurafenib	NDA202429	P110020	PCR
V600E/K	Melanoma	Vemurafenib	NDA202429	P170021	NGS
V600E	Melanoma	Dabrafenib	NDA202806; NDA 202806/S-006	P170021	NGS
V600E	Melanoma	Trametinib	NDA 204114; NDA 204114/S-005	P170021	NGS
V600E	Melanoma	Trametinib	NDA 204114; NDA 204114/S-006	P160045	NGS
V600E	Melanoma	Dabrafenib	NDA202806/S006	P160045	NGS
17p−	B cell chronic lymphocytic leukemia	Venetoclax	NDA208573	P150041	FISH
ROS					
ROS1 fusion	NonSmall Cell Lung	Crizotinib	NDA 202570; NDA 202570/S-021	P160045	NGS (RNA)
Isocitrate dehydrogenase-2					
IDH2 mutations (R140Q, R140L, R140G, R140W, R172K, R172M, R172G, R172S, and R172W)	Acute myeloid leukemia	Enasidenib	NDA209606	P170005	PCR
FLT3					
FLT3 D835 and I836 mutation	Acute myeloid leukemia	Midostaurin	NDA207997	P160040	PCR

*IHC* immunohistochemistry, *FISH* florescent in situ hybridization, *RTPCR* reverse transcriptase polymerase chain reaction, *T790M* threonine to methionine mutation at amino acid position 790 in human epidermal growth factor receptor, *D816V* aspartic acid to valine mutation at amino acid position 816 in human epidermal growth factor receptor

The HGP, in and of itself, is a kind of “controlled” of “A vs. B” experiment in comparing proprietary vs. open innovation. There was a private, proprietary project led by Celera Genomics in parallel with the publically funded project [4]. Two academic bioinformaticists, Weber and Myers, devised a strategy for in silico reassembly/alignment of genetic sequence fragments (the “shotgun approach”) that utilizes the localization of long interspersed nuclear elements (LINEs) and nonrepeating sequences [5]. That strategy became a key element driving Celera’s high throughput genome sequencing approach (referred to as the private human genome project). The public, NIH funded HGP, was a critical catalyst for the creation of Celera and much of the sequencing technology that was developed by multiple genomics tools companies and used during the HGP and thereafter.

The HGP has impacted biology, medicine, and agriculture in transformative ways with multiple intellectual spillover effects [6]. The use of alignment technology was a pivotal milestone in the HGP and exemplifies academic science, open innovation, and public private partnership having significant social impact and major ripple effect 15 years later and beyond. Notably, this innovation was not constrained by the Bayh Dole Act and was used freely by a vast swath of science ranging from graduate students doing BLAST alignments to the senior scientists responsible for the strategic underpinnings of the HGP.

Open innovation is a model and ethos for knowledge and technology dissemination that essentially strives to expand the “knowledge commons” of an innovation ecosystem in a manner that might move a company, industry, or an innovator ecosystem forward. This has been studied and reviewed extensively by Henry Chesbrough [7]. A fundamental premise of open innovation is that inter-firm knowledge transfer can accelerate R&D. In industries where complexity and a diversity of capabilities, and specialized infrastructure are required to bring a solution to market, open innovation is touted as a business method where channels of external cooperation can be synergistic. Healthcare is such an industry.

Myer’s contribution to the field of computational biology certainly exemplified the open innovation model and ethos, though the underlying proprietary genomic annotation business model of Celera was intended to be profitable. Shotgun sequence reassembly technology shared with the participants of the HGP was arguably a major catalyst that dramatically accelerated completion of the project and the massive private investments in genomics application that were a direct result of the new human genome data ecosystem [1, 5, 6]. While it is argued by some economists that the dollars actually deployed as salaries and direct costs for the HGP were costs, and not benefits [2], we posit that the spillover of human capital, expertise and experience, resulting from those expenditures have been an impactful driver of the precision medicine innovation ecosystem in AMCs.

Shotgun sequence alignment technology is an apt corollary to the application programming interface (API) for the human genome, its release into the wild spurred a wave of innovation that unarguably had positive economic impact. Despite much rhetoric and hysteria at the time about the human genome being patented, privatized, and profiteered, the highest order use of the knowledge the HGP enabled, has happened at the academic-industry interface in the context of using sequencing to identify the molecular determinants of disease and response (or nonresponse) to therapy. Molecular interrogation of indicators of response to therapy, and differential diagnosis are the next commercially tractable frontier in genomics.

In the initial years following completion and publication of the HGP, the complex information from reference genomes was rarely useful or actionable in the clinic; despite being incredibly useful to the research community. This challenge largely remains at press time, though progress has been made in using somatic sequencing in oncology [8] and germline sequencing [9] in differential diagnosis of rare disease. Research strategies have quickly refocused on addressing this gap in understanding the clinical implications of genetic differences, accelerating investment in translational research, and enabling the field of precision medicine [10]. The genomic revolution spawned countless innovations, both public and proprietary, several drugs targeted at disease with a genetic component (Table 1), a vast commercial ecosystem of sequencing and bioinformatics companies, and at least one US Supreme Court patent case [11]. Importantly, the HGP spawned both hype and endless hope, some realized, and most still pending [12].

### The clinical leap, the population paradox in personalized care, and commercial realities

The raw ore of genetic information (at least as it relates to select reference genomes) is now readily abundant, and by and large, in the public domain. Soon, genome and transcriptome sequences (and accompanying clinical annotation) will be easily obtainable at the individual/population level and used in the course of routine individualized medical care. However, a question that still remains: how actionable is this sequence information? The clinical annotation of raw genetic information has proven at least as important and useful as the founders of Celera imagined when they predicated their business model on a molecularly annotated version of the publically available reference genome sequence. Interestingly, the clinical annotation of the data is perhaps the most interesting and relevant information, a broad molecular understanding of disease by the physician at the clinical case level is necessary to reap the envisioned rewards of the HGP. Consequently, AMCs have prioritized investment in specimen banks and clinical data warehouses to accumulate data, an increasingly important currency in collaboration between industry and AMCs. The ability of physician scientists and clinical research specialists to toggle between clinical outcome data and genetic information has been enabled by commercially available dashboards that summarize clinically relevant elements of a clinical sequencing report; though, many physicians remain less interested in receiving information that may not be actionable, may be confounding, or create risks of backward looking malpractice claims. Consequently, dashboard reports with only actionable mutations are the norm in most commercially available sequencing tests.

Population level sequencing is a priority that has become a critical element of many clinical research projects at AMCs and industry sponsored drug registration trials. Thus, companies are investing in “sequencing factories” to enable quasi-proprietary population level sequencing [13]. The power of mining the electronic health record annotated with personalized genetic information should not be underestimated. Our collective capacity to understand the molecular basis for disease by in silico hypothesis formulation and testing and by using existing data as training sets has been enhanced orders of magnitude relative to the times before the HGP was first published because analysis of the polymorphic variations that define the genetic basis of disease are now possible [14].

Progress has been most visible in translational oncology, where genetic information has been sought and applied more aggressively, to become more actionable than other disease areas like neurodegenerative or cardiovascular diseases. Over 2.5 M oncology companion diagnostic tests were performed in 10 major markets in 2016 [15]. Many more molecular diagnostic assays are available as laboratory developed tests (LDTs under the Clinical Laboratory Improvement Amendments), analyte specific reagents for prognostic value or as supplemental diagnostic information, with the growth of the list of reimbursed molecular tests growing as the clinical literature supports the use of emerging biomarkers. As of press time, 38 FDA-approved molecular in vitro diagnostic tests were identified, canvasing 113 genes and 85 actionable analytes, with evidence-based decision rules enabling the test-then-treat paradigm for 26 different drugs (Table 1). The European Medicines Agency has not historically connected drug and in vitro devices, thus currently lacks a comparable framework for companion diagnostic approval to the FDA. In July 2017, the EMEA released a concept paper in the spirit of establishing regulatory coupling and alignment of companion diagnostic development with clinical drug development, in essence a provision of guidance for obtaining a CE-mark on a diagnostic assay that is a companion diagnostic [16].

In a detailed analysis, Scannell et al. [17] showed that R&D productivity (as measured by the number of approved drugs) has declined along a somewhat predictable trend deemed Eroom’s law (Moore’s Law, spelled backward). The reasons for this decline are likely complex and varied; Scannel et al. propose four causes for the downward trajectory–cautious regulation, increasingly difficult comparators in existing drugs, indiscriminate investment, and assuming clinical success is correlated with the number of compounds screened. It is widely accepted that failed clinical trials, particularly Phase 3 trials, are a major cost driver contributing to the decline in most measures of R&D productivity. So why has R&D productivity declined as the price of sequencing has fallen to under $0.01 per megabase [18]? We posit here that as the design of drugs becomes more focused on the target and the molecular basis of disease, there is increasing statistical noise caused by enrollment of patients lacking the molecular profile that makes them responsive to a targeted treatment approach. The FDA issued a whitepaper in 2004 that culminated in identification of clinical drug development challenges (and failure points) that could be addressed through rigorous biomarker qualification, with significant academic participation in those efforts, including the establishment of the Critical Path Institute [19]. The reproducibility of experimental findings across different laboratories, coupled with limitations extrapolating from animals to humans, have been major impediments to translation of academic research, particularly in drug development [20]. The heterogeneity of disease in human populations, along with classifications of disease based on symptoms, histology, and historical descriptors, have collectively contributed to failed clinical trials, despite promise in animal studies. The trend toward identification of the molecular underpinnings of disease, coupled with efforts to develop treatments better aligned with objectively measured molecular defects, is likely to circumvent many flawed assumptions about both the relevance of animal models and the likelihood of patient responses to therapeutic interventions.

One approach to avoid clinical development of drugs that fail efficacy trials is early and rigorous validation of biomarkers that might be used to guide enrollment and become the basis for companion diagnostic assays. Peer reviewed publication of clinical research on biomarkers is likely inadequate as the AMC research enterprise and peer review process do not apply all of the rigorous quality standards that industry and FDA ultimately require for the use of a candidate biomarker for therapeutic decision making. The coordination of robust assay development arguably is best done with an industry diagnostic partnership, early in drug development, such that regulatory, legal, quality, and commercial considerations are factored into the regulatory science and development plans in the earliest clinical studies. The need for development of diagnostic tools early in drug development is a major driver of the rate of change of partnership models among biopharma drug developers, AMCs, and diagnostic technology companies.

Gefitinib was originally approved by the FDA in 2003. In 2005, results published in the Lancet from a pivotal study of gefitinib in nonsmall cell lung cancer patients (ISEL trial) showed a very positive tumor response in a subset (13%) of patients, but the overall survival benefit in the enrolled Caucasian population was not statistically significant [21], consequently the FDA reversed the approval. By 2006, the race was on to identify biomarkers that would allow prospective identification of the responsive subset of patients [22–24]. Mutations of the epidermal growth factor receptor (*EGFR*) gene were the leading candidate biomarker [22–28]. In 2011, the American Society of Clinical Oncology advanced clinical guidelines recommending the use of *EGFR* mutation testing in non-small cell lung cancer (NSCLC) patients in order to consider *EGFR* tyrosine kinase inhibitors as first line therapy [27]. While widely used outside the US in the interim, gefitinib was not used regularly to treat lung cancer in the US. Erlotinib was approved and widely used to treat lung cancer after 2004. In July 2015, 10 years after the initial pivotal trial results were published, gefitinib was approved by the FDA for front line treatment of NSCLC positive for *EGFR* exon 19 deletion or L858R mutation in exon 21 [28], contemporaneous with the companion diagnostic assay kit that was used in its pivotal registration (Table 1, Food and Drug Administration PMA #P120022). It is notable this approval was designated as an orphan indication. Gene copy number and protein expression level [23] also showed some potential for use as biomarkers for EGFr tyrosine kinase inhibitors in lung cancer, but never gained the level of usage of EGFr mutation analysis, as was the case for Her2 in breast cancer clinical management and the use of Her2 targeted therapies.

### Academic medical centers and genomic medicine

AMCs, along with their translational research infrastructure (CAP-CLIA genomic labs, specimen banks, and clinical data warehouses) will increasingly be a critical element in innovative drug development projects that require discovery of, or analysis of biomolecular determinants for clinical trial enrollment.

Genome plasticity and instability are increasingly being recognized as targets for cancer therapies and consequently, there is a greater role for the AMCs, and their key opinion leaders, in defining polymorphic molecular targets for use as enrollment criteria. Cancer care in recurrence cases and metastatic disease is increasingly reliant on defining genetic changes relative to the original primary tumor, preferably using a liquid biopsy specimen [29]. Liquid biopsy is a promising diagnostic tool, and AMCs are increasingly important as sources of specimens and longitudinal outcome data that can help define the clinical utility of liquid biopsy assays. The molecular defects that are targets for new therapeutic strategies like chimeric antigen receptors in engineered T cells, are only possible in an environment where the molecular basis of disease, and the biological engineering of the therapeutic intervention, can be established in close collaboration with the attending physician at the point of care. As such, AMCs will be at the forefront of innovative new approaches to biomarker guided and stratified drug development approaches. A common thread is that AMCs play a central role in observing, validating, and defining the clinical decision rules that emergent biomarkers can enable. As innovation and validation cycles for emergent biomarkers shorten, AMCs are increasingly a major contributor of the peer-reviewed science that clinical sequencing companies rely upon to drive the clinical use cases for their panels and assays underlying their business.

Assays for specific menus of mutations are increasingly commoditized and decreasingly proprietary. The currency of a patent is not as valuable to AMCs as it once was in engaging diagnostic companies. In contrast, a robust biobank linked to the clinical data ecosystem has now become valuable currency as clinical evidence becomes an increasingly important market driver for new diagnostic business models that use next generation sequencing and other high throughput technologies for diagnostic medicine. Reimbursement, patent, and market landscape dynamics for in vitro diagnostic kits will continually erode the proprietary value of an assay kit [30]. There is a trend toward open dissemination of knowledge supporting biomarkers validation and peer review of biomarkers used in the course of clinical trial stratification and in the treatment decision making process itself [30]. These trends further the commoditization of biomarkers as content (as demonstrated by the recent approval of Foundation 1 CDx, Table 1), There are a number of biobank networks, registries, and data ecosystems emerging in oncology that are aggregating clinical cases and –omic data at population scale. Some are primarily academic consortia, others are mostly supported by private companies. Cutting-edge computational approaches like machine learning can be applied to mine and infer critical information from real clinical cases where clinical and outcome annotation are coupled with sequencing data.

The clinical validation of many cancer biomarkers has predominantly been supported financially by biopharma companies and the NIH. Diagnostic companies lack the capacity and interest in funding prospective clinical validation studies. This is in part because of the Appropriability Conundrum [31] (that is, all diagnostic market participants generally benefit when a biomarker is validated) and in part because of practical limitations in using the patent system to compete in the diagnostic marketplace [30].

Another challenge for AMCs is accelerating technology obsolescence cycles. The capital expenditure decision processes of academic institutions make it difficult for institutions to place bets on all possible emerging technologies and scale infrastructure to become a market competitive service provider. However, diagnostics companies are increasingly better positioned to assume the capital expenditures and operating costs of specialized industrial scale-and-quality analytic technologies than AMCs, and an emerging collaboration model is one where the company analyzes specimens from an AMC and the clinical insights are shared between the two. This is a more capital efficient model for AMCs to seek emergent biomarkers using the latest analytic technology without necessarily making an immediate capital outlay. The diagnostic company often gets peer-reviewed publications validating a commercially relevant use case for their technology, and perhaps access to “proprietary content” (novel biomarkers, reagents, and assay formats) though patenting diagnostic decision rules and biomarkers encounters the difficulties reviewed later.

The affiliations between academic and healthcare entities, and their joint governance is an important determinant of how such collaborations happen. The University of Arizona and Banner Health entered into an affiliation in 2015 in part, to leverage opportunities to advance precision medicine by virtue of aggregating population level data and specimen resources [32]. A constructive interaction between clinical and research enterprise is critical to utilize these powerful assets, and the human element of these systems interact should not be underappreciated.

### Genomic medicine and technology transfer

The trend toward analyte consolidation has implications for traditional technology transfer practices at AMCs and is fundamentally changing the texture of the academic-industry interface in clinical research and diagnostic medicine [30]. Many molecular biomarker reagent innovations (probes for mutations, probes for genetic rearrangements, probes for deletions, or reagents that alter the expression of proteins or mRNAs), fit into a traditional business and revenue model in the diagnostic industry: selling assay kits and providing CLIA lab services. Assay kits are likely to decrease in prevalence as sequencing costs drop and high-throughput biology forces single analyte and low-content in vitro assays to become obsolete. Clinical diagnostic kits and reagents are becoming less compelling medical innovations for the traditional “patent and license” model of technology transfer, as content becomes harder to own (i.e. to patent). The market for molecular probe products and simple in vitro assays is being disrupted by a market that caters to high throughput platforms, interpretative tools and ‘omics services that are useful in making clinical decisions around the genome sequence of a patient, their diseased tissue, or transcript profiles in diseased tissues. Eventually, a majority of sequence based tests will utilize highly specialized and differentiated algorithms that seek genetic patterns, whether single SNPs, or gene expression patterns based on a complete genome, transcriptome, or proteome (versus a targeted subset thereof).

Recent patent case law and legislation have done little to elevate the role for patents and traditional patent licensing in biomarker commercialization, particularly in cases where market forces favor multianalyte assays [30]. Multianalyte tests increasingly enabled a menu of clinical decisions, as illustrated by the recent approval of Foundation One CDx which is a next-gen sequencing (NGS) assay that detects defects in 324 target genes and can guide the use of 18 drugs for which it is approved (Table 1). NGS ultimately allows detection of most of the known disease-related genetic variants (though many still are not clinically actionable) in a sample of the relevant genetic material, and takes advantage of computational approaches with robust bioinformatics tools [33]. Importantly, data analytics are enabling the identification and linkage of suspected pathogenic variants to disease using sequence data derived from static genetic material (somatic or original tumor) and in silico analysis of outcome data from clinical cases aggregated from electronic health records. In some cases, liquid biopsy technologies may allow sequential comparisons of outcome data to a dynamic genetic analyte. In essence, artificial intelligence technologies can be applied iteratively across time and longitudinally throughout the natural course of disease to correlate genetic variants with progression, severity, risk, drug responses, and other outcomes. These iterative roundtrips of analysis are largely occurring outside the context of a traditional managed clinical trial, so these undertakings require a thoughtful approach to consent, protect privacy, de-identify subjects, and contract data and IP rights with clinical trial sponsors, healthcare affiliates, and vendors in the health data and specimen supply chains. Due diligence on chain of custody of specimens, consents, and data at the patient level is impractical, and in many cases impossible. Such approaches to clinical research, biomarker discovery, and biomarker validation, make the origins of data and specimen ownership extremely diffuse. That in turn makes attribution of IP rights even murkier, in that a data set leading to an invention or reduction to practice come from multiple sources and the chain of custody on such data can be impractical or impossible to document to its source. The diffuse attribution of outcome and biomarker data commons in most clinical research environments further justifies an open innovation approach to biomarker discovery, especially when considering how most population level ‘omics data sets are generated today. Sources of genomic and clinical data are diffuse and this reflects the reality that there always is some moderate degree of probability that a biomarker patent by an AMC contains data or relies upon clinical cases that were not perfectly documented. Blockchain technology represents a potential solution as an immutable transaction ledger, perhaps easing the exchange of personal health information and specimens [34] Consequently, a paradox has emerged for the AMC trying to engage industry partners to advance biomarker IP. On one hand, there is a more crucial role in population-level biomarker discovery, validation, and clinical deployment of new analytic technology. On the other, there is a more limited ability to own, protect, and therefore participate in commercial outcomes by enabling products and royalties.

It is difficult for a diagnostic company to recover their investment in clinical trials for a variety of reasons, including low barriers to regulatory entry for concordant assay methodologies. It is also challenging to own significant space around a certain biomarker or biomarker class. This is evidenced by the broad spectrum of analytes and assays in companion diagnostics identifying *Her2/Neu* and *EGFR* defects (Table 1). Reimbursement models for molecular diagnostics and multianalyte tests are in need of revision, and some progress has been made in the establishment of reimbursement rates that reflect both the cost and value of certain genomic assays. However, low reimbursement coupled with fragmented markets around a biomarker or clinical use case makes the investment thesis tenuous for diagnostic companies considering prospective clinical studies. The reality is that genomic content is likely to be validated organically through the evolution of a knowledge commons and years of peer reviewed publication using large populations, as cholesterol and homocysteine gained clinical adoption in cardiology. However, one can no longer get broad patent protection on diagnostic content like homocysteine [30, 35]. Commercial value of diagnostic content is not a driver of collaboration under the traditional technology transfer paradigm given the devaluation of diagnostic patents, however, validation data sets and clinical cases are increasingly valuable to industry.

In parallel to legal and market factors that have reduced interest and valuation of academic biomarker patents, academic institutions have increasingly been driven by stakeholders to license technology to spinout companies [36]. Startup companies are a more direct and visible return on research investment for an AMC and grantor, than technology that is licensed, often confidentially, into a large corporation. Consequently, there has been a general (not specific to diagnostic technology) uptick in the number of startup companies as a means for academic institutions to demonstrate and effect clinical and societal impact in a manner highly visible to stakeholders. These are still arm’s length transactions between academic institutions and startup companies. The financial terms of these transactions generally require development of milestone payments and sales royalties payable to the university who owns the underlying intellectual property. Diagnostic innovations are unique in that many AMCs have embedded clinical diagnostic enterprises including pathology laboratories, molecular testing, and medical imaging that can represent market channels for nascent diagnostic technology under existing regulatory frameworks like laboratory developed tests. In these instances, the intellectual property is commercially deployed within the institution, thus interesting questions of intrainstitutional allocation of technology value arise. For example, would the pathology department or a hospital affiliate be required to license a patented assay or reagent, or pay a royalty to the technology transfer office? Which units would bear financial responsibility for prosecuting and enforcing patents? Is exclusive licensing and offering from an academic testing enterprise consistent with the public-serving mission of a public university? Which commercial technology channel is most aligned with an institutions’ mission and stakeholder expectations? Large diagnostics licensee? Spinout company? Internal testing enterprise? It is possible that an assay or novel biomarker needs to be offered as a CLIA test by an academic CLIA lab in order to achieve a degree of clinical validation and potential reimbursement. In summary, many forces are altering and complicating the ultimate commercial disposition of proprietary reagents or assays that originated from AMCs. These new and complex market forces raise unique considerations for an AMC, relative to medical devices and therapeutics. Well-capitalized external companies are the only ultimate pathway for delivery of drugs and therapeutic devices to customers and end users, however many AMCs have enterprises that can deliver diagnostic content directly to physicians and patients. The primary impetus behind these diagnostic enterprises is not revenue per se, rather the needs of the healthcare and research enterprises of the AMC. Measuring novel biomarkers where assays might not yet be commercially available is certainly a major benefit of having these diagnostic enterprises. Increasingly, having the raw genetic data to augment clinical case data is a valuable capability for AMCs in their efforts to build and leverage the valuable data ecosystems inherent in their clinical enterprises.

### Genomic medicine and big data collaboration models

A number of clinically oriented laboratories and analytics companies have reduced the cost of sequencing whole exomes and whole transcriptomes to a price point within the reach of the patient and the clinician. Whole genome sequencing is fast becoming practical at the clinical case level based on the rate of change in cost per megabase, which is a cost reduction curve much steeper than Moore’s Law, which postulates that technology reduces microprocessor costs by half every 18 months. Going forward, clinical value will increasingly be created at the point-of-care in the delivery of interpretation (decision tools and rules) of complex information and artificial intelligence (AI) assisted clinical decision-making. AI, as applied to clinical decision-making is already used with waveform physiological data in an ICU setting [37]. Soon, similar AI-based business models using ‘omics data sets are expected to emerge to exploit the knowledge and information AMCs and healthcare providers are accumulating in our nation’s clinical data warehouses. IBM currently offers services under its Watson Health brand utilizing natural language process and other AI techniques to utilize genomic data, patient electronic health record, case level clinical notes, and paradoxically, the universe of relevant scientific literature, to recommend to physicians stratified menus of differential diagnoses, therapies, and comparable cases. GE made a major investment in defining medical use cases for its Predix Health Cloud internet of things platform, an application ecosystem for applying AI and distributed computing technologies to draw inferences from electronic health record, sensor, and image data [38].

The HGP has been transformative in enabling the molecular diagnostics industry to rise from the human genome, but rapid and disruptive innovation in high-throughput biomolecular analytics is pushing biomarkers out of the realm of proprietary kit and in vitro assay productization, and into the realm of open innovation. The new high-content paradigm is likely to enhance the role of the AMC in bridging diagnosis and treatment resembling the software-as-a-service (SaaS) business model. This trend is simply an acceleration of the technology driven evolution of medical practice to deeper specialization, with the relevant practitioners, and the patients that require care from them, gravitating to the infrastructure-rich environment of AMCs.

There is substantial opportunity for the AMC to help the healthcare industry overcome the interesting paradox that applied genomics and precision medicine present, the” n-of-1” challenge. At the molecular level, every clinical case is somewhat unique, so scalability and statistical power in a clinical study can be elusive, especially when trying to accrue a clinical trial within a single institution. The solution lies in the melding of practices from academia (creating data commons through consortia) and industry (quality standardization in clinical protocols). The collection of specimens and health information from vast numbers of subjects in many institutions under high-quality and standardized protocols is increasingly important.

The Cancer Genome Atlas is among the first publically funded efforts at population scale genomic sequencing in oncology. Shortly after its launch, The Oncology Research Information Exchange Network (ORIEN) and the use of the Total Cancer Care Protocol represented promising approaches to address challenges of precision medicine research [39]. The ORIEN consortium of collaborating top cancer centers has enabled collection of over 100,000 carefully consented, clinically annotated tissue and genetic specimens to date. The more recent Precision Medicine Initiative *All of Us* research program has been designed to collect environmental, lifestyle and genetic information on over 1 million participants throughout the US in order to advance precision medicine research and practice, with major AMCs collaborating throughout the country to enroll participants and facilitate research. The All of Us program will also require the ecosystem of participants to find solutions to the many daunting challenges in creating large data ecosystems: a flexible electronic consent, data sharing in a “cooperative” context, large scale enrollments, finding genetic diversity, and legal and regulatory administration.

Several for-profit organizations, Paradigm Diagnostics, Flatiron Health, Foundation Medicine, Curis, Tempus, Syapse, and M2Gen, are creating consortia of academic research and medical centers to aggregate clinical cases and create registries, in a manner similar to OREIN, at population scale (with aims to accumulate tens of thousands of cases). The business models their data ecosystems enable are unique, and collectively diverse, and a topic worthy of deeper discussion, but beyond the scope of this review. A common theme in their business models is that their efforts to build large-scale data sets enable an unprecedented opportunity for cancer and precision medicine researchers to zoom into the individual and then zoom out to the population to test hypotheses in silico with vast access to hundreds of relevant data points in retrospective data sets. There are a myriad of legal, practical, and privacy hurdles to achieving the data ecosystems these firms strive to develop.

AMCs have a definitive scale advantage, and will be able to contribute substantively to these data commons, while playing a role in organizing and curating valuable resources specifically for the benefit of the scientific and clinical communities. However, the most important role that differentiates AMCs from private sector participants like the diagnostic and pharma companies, is that AMCs is new healthcare delivery models and validation of new decision rules can easily take place.

The clinical and commercial innovations stemming from the data commons projects in this review are expected to be transformational to medicine, and to catalyze the transition from evidence based medicine to algorithm based medicine [40–42]. It is expected that physicians will have ready access to the practical tools, decision rules, and dashboards to address the heterogeneity of disease and fully realize the promise of precision medicine. There is a striking irony that population level ‘omics datasets are finally making individualized healthcare practical.

## Conclusions

Academic medical center leaders and administrators first must recognize and acknowledge the unique requirements of their clinical affiliates and biopharma partners. In precision medicine collaborations, resolution of intellectual property, data rights, and specimen management issues increasing requires a scientific and clinical perspective to augment the abstract legal approaches generally used in research contracting and technology transfer. Establishing clear ownership, or worse, sole ownership, of these research work products is an increasingly difficult source of friction and increased transactions costs. Sharing of these research outputs provides a more flexible approach to balance the various needs of the clinical, academic, and industry participants that are party to most precision medicine collaborations where the data currency is increasingly a common objective of all parties (though different uses are envisioned by biopharma companies, academic investigators, and clinical affiliates). Medical knowledge and clinical case data are an increasingly important dimension of the industry-AMC interface. The administrative processes and institutional strategies of AMCs should acknowledge that clinical cohorts and highly specialized medical expertise are far more differentiated assets than clinical NGS services and biomarker IP, though the latter two assets can augment a strong institutional precision medicine strategy.

## Abbreviations

HGP: Human Genome Project
AMC: academic medical center
LINE: long interspersed nuclear element
NIH: National Institutes of Health
BLAST: basic local alignment search tool
R&D: research and development
API: application programming interface
FDA: Food and Drug Administration
EMEA: European Medicines Agency
LDT: lab developed test
EGFR: epidermal growth factor receptor
NSCLC: non-small cell lung cancer
L858R: leucine to arginine substitution mutation at amino acid position in human epidermal growth factor receptor
CAP: accreditation by the College of American Pathologists
CLIA: Clinical Laboratory Improvement Amendments
NGS: next generation sequencing or sequencing by synthesis
IP: intellectual property
Her2/neu: human epidermal growth factor receptor 2
AI: artificial intelligence
ICU: intensive care unit
SaaS: Software as a Service
PCR: polymerase chain reaction
